# Seed Morphology of *Allium* L. (Amaryllidaceae) from Central Asian Countries and Its Taxonomic Implications

**DOI:** 10.3390/plants9091239

**Published:** 2020-09-20

**Authors:** Shukherdorj Baasanmunkh, Jae Kyoung Lee, Ju Eun Jang, Min Su Park, Nikolai Friesen, Sungwook Chung, Hyeok Jae Choi

**Affiliations:** 1 Department of Biology and Chemistry, Changwon National University, Changwon 51140, Korea; Baasanmunkh.sh@gmail.com (S.B.); nosejk@naver.com (J.K.L.); jueunjang222@gmail.com (J.E.J.); 2 Department of Biology Education, Kongju National University, Gongju 32588, Korea; lacmyo14@gmail.com; 3 Botanical Garden of the University of Osnabrueck, 49076 Osnabrueck, Germany; friesen@biologie.uni-osnabrueck.de; 4 Department of Computer Engineering, Changwon National University, Changwon 51140, Korea; swchung@changwon.ac.kr

**Keywords:** *Allium*, seed morphology, seed testa, taxonomic significance, central Asia

## Abstract

We studied seed macro- and micro-morphological characteristics of 48 *Allium* species (51 accessions) belonging to 24 sections and 7 subgenera. Our taxonomic sampling focused on the central Asian regions of Uzbekistan, Kyrgyzstan, and Mongolia. The seed length ranged between 1.74 ± 0.16–4.47 ± 0.43 mm and width ranged between 1.06 ± 0.08–3.44 ± 0.23 mm, showing various shapes. The irregular and elongated polygonal testa cells occurred in all investigated species. Seed testa sculptures showed high variation in their anticlinal walls associated with different shapes: straight to with U-, S- or Omega-type undulations among the species. The moderately flat to convex periclinal walls with various sized verrucae or granules were found in all investigated taxa. Based on our research, we conclude that seed characteristics such as size, shape, and the seed testa features show their significant variability, revealing key characteristics to support taxonomic relationships and major clades recovered in the molecular phylogeny of the genus *Allium*. Especially, the anticlinal wall characteristics were highly variable and decisive at the both section and species levels. In addition, widely varied shapes and sizes of the seeds were remarkably effective to distinguish *Allium* species.

## 1. Introduction

The genus *Allium* L., comprising more than 920 species [1], is one of the most diverse and the largest genus of monocots [2,3]. The most recent classification of the genus accepted about 800 species belonging to 15 subgenera and 72 sections [2]. Li et al. [3] defined 13 subgenera and 34 sections based on morphological and molecular studies of *Allium* in China. Recently, some ambiguous subgenera and sections have been revised, depending on their morphological characteristics, molecular information, biogeographical distribution, and evolutionary history. In particular, the partial revisions in sect. *Cepa* [4]; subg. *Melanocrommyum* [5,6,7]; subg. *Amerallium* [8,9], subg. *Cyathopora* [10]; sect. *Oreiprason* [11]; subg. *Anguinum* [1]; sect. *Rhizirideum* [12], and sect. *Rhizomatosa* including sect. *Caespitosoprason* [13] were lately conducted.

Seed macro- and micro-morphology have been suggested to taxonomically distinguish the species and sections of the genus *Allium*. There are some fundamental studies on seed testa characteristics in genus *Allium* species, investigated by Bothmer [14], De Wilde-Duyfjes [15], Pastor [16], Kruse [17,18,19,20], Češmedžiev and Terzijski [21], and Ilarslan and Koyuncu [22]. More comprehensive studies on the seed sculpture and molecular information have been presented; Fritsch et al. [23] investigated seed micro-morphology of *Allium* species form the Middle Asian countries. Similarly, Neshati and Fritsch [24] examined the seed sculptures of 20 species from Iran. Choi and Cota-Sánchez [25] researched the seed sculptures of *Allium* in Canadian prairie regions. Moreover, Choi and Oh [26] studied key morphological characteristics including the seed shapes of 24 species of *Allium* in Korea and northeastern China. Bednorz et al. [27] described seed characteristics of eight *Allium* species from Poland. Celep et al. [28] investigated both seed size and shape with testa characters of 62 species and Duman et al. [29] studied seed testa of six species in Turkey. According to Choi et al. [30], the seed testa sculpture attributes in combination with seed shape provide key characteristics to distinguish major clades in the molecular phylogeny in northeastern Asian and northern North American *Allium* species. Recently, Lin and Tan [31] studied seed coat characteristics of 39 species that belong to 19 sections of *Allium* in China (Xinjiang) and divided them into six groups. In addition, Veiskarami et al. [32] investigated seed size and shape as well as micro-morphology of 23 species belonging to two subgenera and six sections in Iran. Currently, several new taxa have been recognized in this genus based on their morphology including seed macro- and micro-morphological characteristics and phylogenetic studies [29,33]. Some other characteristics that provide important taxonomic information in *Allium* for infrageneric groups are anatomy of bulb tunic, scape, and pollen morphology [29,34,35,36].

The central Asian countries, particularly Uzbekistan, Kyrgyzstan, and Mongolia, have high diversity of *Allium* species. For instance, the estimated number of *Allium* species in Uzbekistan, Kyrgyzstan, and Mongolia is 195, 85, and 52, respectively [37,38,39,40,41,42]. In Mongolia, most of the species had been studied comprehensively by their morphology, cytology, geographical distribution, ecology, and molecular phylogeny [12,37,40]. However, studies on seed morphology including testa structure are still limited. In the meantime, in Uzbekistan and Kyrgyzstan, only few species’ seed morphology was investigated to date [23].

In this study, we examined seed morphology and testa sculpture of *Allium* species from the central Asian region of Uzbekistan, Kyrgyzstan, and Mongolia. Our goals of the current study are (1) to expand the current knowledge on seed macro- and micro-morphology, and (2) re-evaluate and discuss the taxonomic implications of the seed characteristics traditionally used to identify various infrageneric groups with respect to the molecular phylogenetic hypothesis.

## 2. Results

The seed shape and size, as well as characteristics of seed testa micrographs, were sorted in alphabetical order of subgenus, section, and species names (Table S1; Figure 1, Figure 2, Figure 3, Figure 4, Figure 5, Figure 6, Figure 7 and Figure 8).

### 2.1. Seed Macro-Morphology

**Figure 1:**
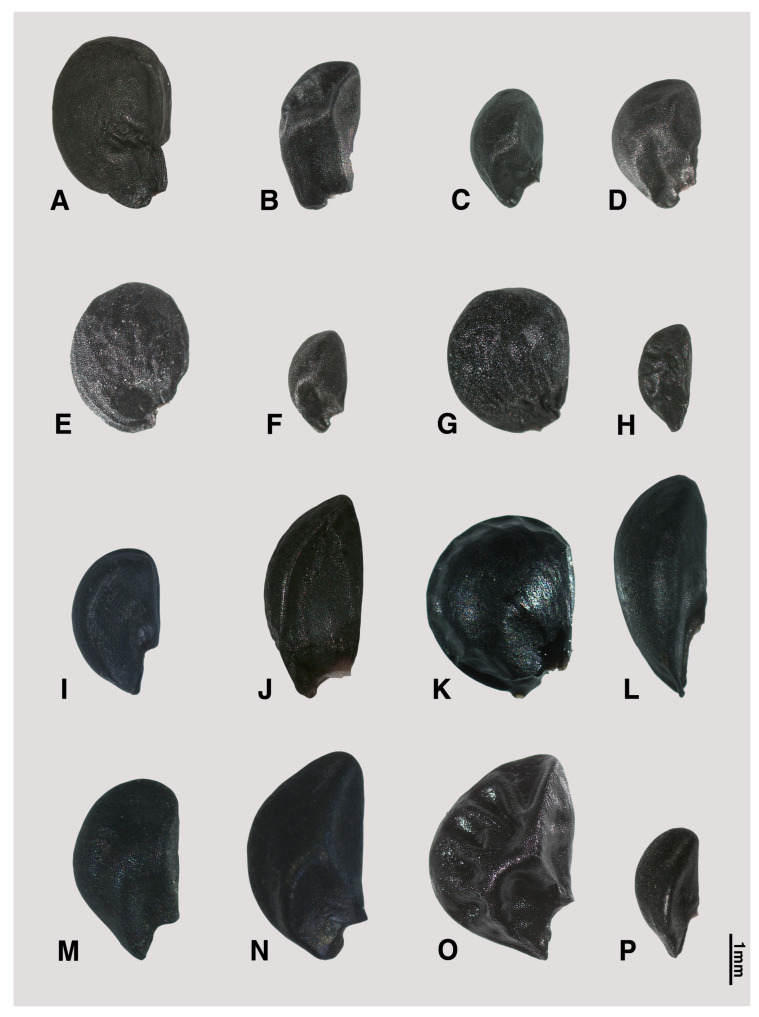
Various shapes of seed in *Allium*. (**A**–**I**) subg. *Allium*: (**A**) *A. filidens* (sect. *Allium*); (**B**) *A. caesium* “101a”; (**C**) *A. caesium* “101b”; (**D**) *A. caeruleum*; (**E**) *A. svetlanae*; (**F**) *A. tatyanae* (sect. *Caerulea*); (**G**) *A. turkestanicum* (sect. *Mediasia*); (**H**) *A. anisotepalum* (sect. *Minuta*); (**I**) *A. pallasii* (sect. *Pallasia*); (**J**,**K**) subg. *Butomissa*: (**J**) *A. oeroprasum* (sect. *Austromotana*); (**K**) *A. ramosum* (sect. *Butomissa*); (**L**–**P**) subg. *Cepa*: (**L**) *A. fedschenkoanum* (sect. *Annuloprason*); (**M**) *A. altaicum*; (**N**) *A. galanthum*; (**O**) *A. oschaninii* (sect. *Cepa*); (**P**) *A. maximowiczii* (sect. *Schoenoprasum*).

**Figure 2:**
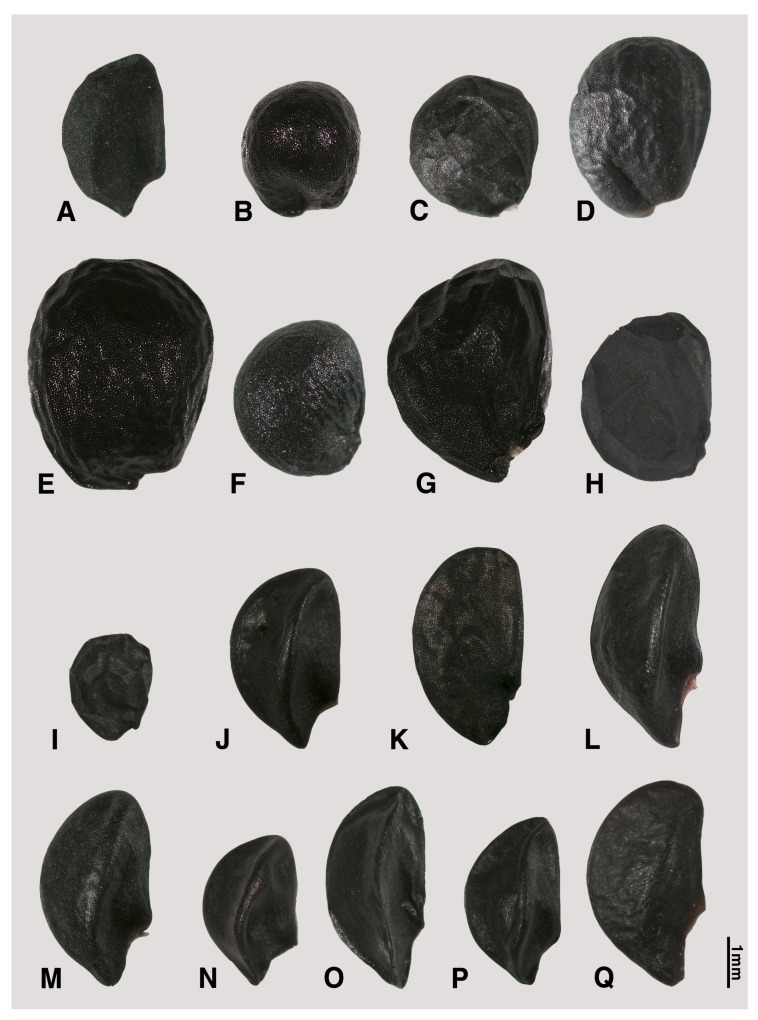
Various shapes of seed in *Allium*. (**A**–**I**) subg. *Melanocrommyum*: (**A**) *A. saposhnikovii* (sect. *Acmopetala*); (**B**) *A. alexeianum*; (**C**) *A. caspium*; (**D**) *A. protensum* (sect. *Kaloprason*); (**E**) *A. karataviense* (sect. *Miniprason*); (**F**) *A. altissimum*; (**G**) *A. stipitatum* (sect. *Procerallium*); (**H**) *A. taeniopetalum* (sect. *Stellate*); (**I**) *A. viridiflorum* (sect. *Verticillata*); (**J**–**Q**) subg. *Polyprason*: (**J**) *A. carolinianum*; (**K**) *A. hymenorrhizum*; (**L**) *A. platyspathum* subsp. *amblyophyllum*; (**M**) *A. platyspathum*; (**N**) *A. korolkowii* (sect. *Falcatifolia*); (**O**) *A. obliquum*; (**P**) *A. petraeum*; (**Q**) *A. tianschanicum* (sect. *Oreiprason*).

**Figure 3:**
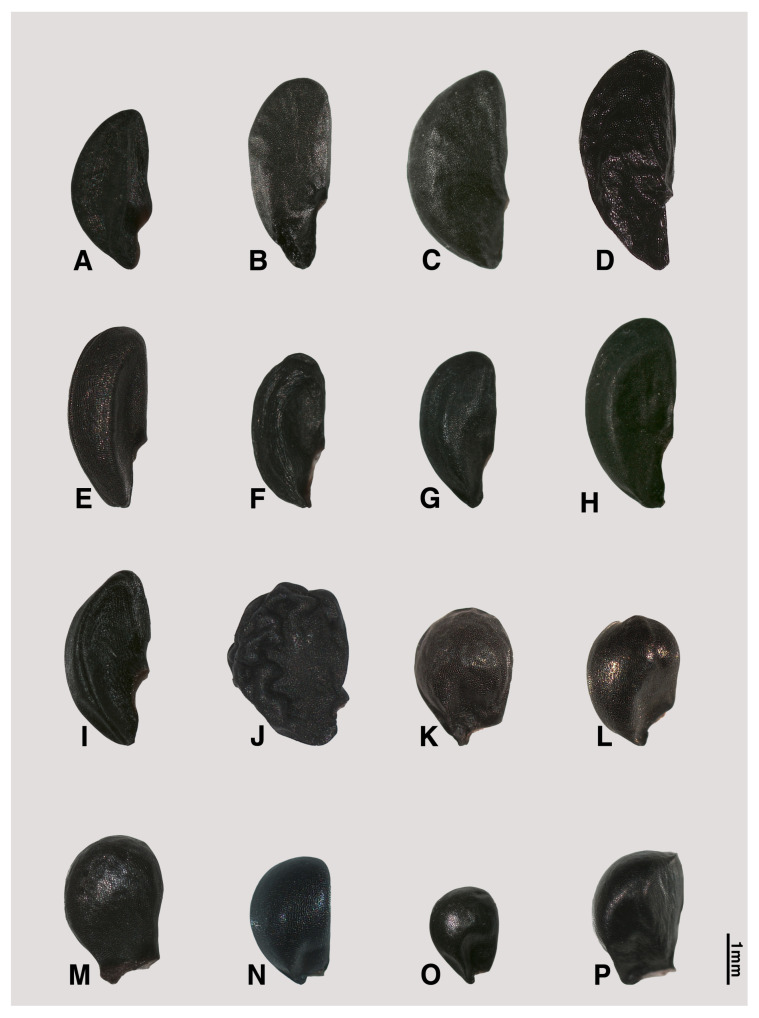
Various shapes of seed in *Allium*. (**A**) *A. kirilowii* (sect. *Falcatifolia*, subg. *Polyprason*); (**B**–**J**) subg. *Reticulatobulbosa*: (**B**) *A. barsczewskii*; (**C**) *A. dolichostylum*; (**D**) *A. jodanthum* (sect. *Campanulata*); (**E**) *A. amphibolum*; (**F**) *A. clathratum*; (**G**) *A. leucocephalum*; (**H**) *A. malyschevii*, (**I**) *A. strictum* (sect. *Reticulatobulbosa*); (**J**) *A. trachyscordum* (sect. *Scabriscapa*); (**K**–**P**) subg. *Rhizirideum*: (**K**) *A. bidentatum*; (**L**) *A. polyrhizum* (sect. *Caespitosoprason*); (**M**) *A. austrosibiricum* (sect. *Rhizirideum*); (**N**) *A. anisopodium*; (**O**) *A. tenuissimum*; (**P**) *A. vodopjanovae* (sect. *Tenuissima*).

The colors of the investigated seeds are black or almost black, while the shapes and sizes are varied in our studied species (Table S1; Figure 1, Figure 2 and Figure 3). Types of seed shapes were categorized as oval-flattened, oval-angular, oval-hemispherical, oval-spherical, elliptical-flattened, or elliptical-angular. According to our morphometric measurements, the seed length ranged between 1.74 ± 0.16–4.47 ± 0.43 mm and the width ranged between 1.06 ± 0.08–3.44 ± 0.23 mm. The longest seed was found in *A. karataviense* (4.89 mm; Figure 2E) and the shortest seed was in *A. tenuissimum* (1.19 mm; Figure 3O). The narrowest seed was found in *A. anisotepalum* (0.83 mm; Figure 1H) and the widest seed was observed in *A. karataviense* (3.88 mm; Figure 2E). The greatest length/width ratio was found in *A. clathratum* (2.31 ± 0.20, elliptical; Figure 3F) and the lowest ratio was in *A. altissimum* (1.13 ± 0.06, oval; Figure 2F).

### 2.2. Seed Testa Sculptures

**Figure 4:**
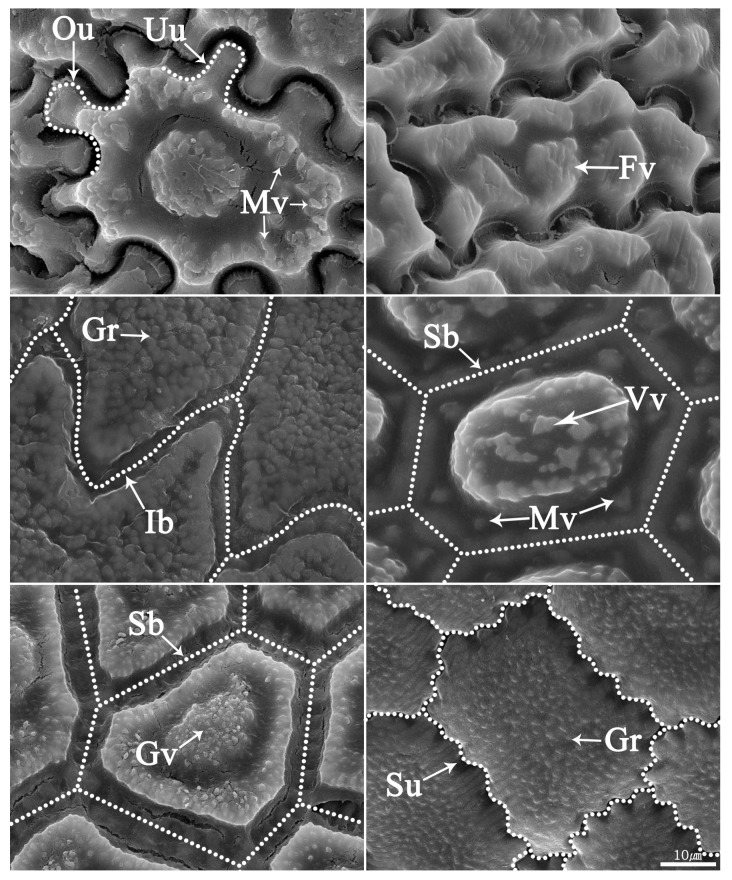
Type of representative features of the seed testa in *Allium*. Ou, Omega-type undulation; Uu, U- type undulation; Su, S- type undulation; Ib, irregular boundary; Sb, straight boundary; Gr, granule; Vv, verrucate verruca; Mv, marginal verruca; Gv, granulate verruca.

**Figure 5:**
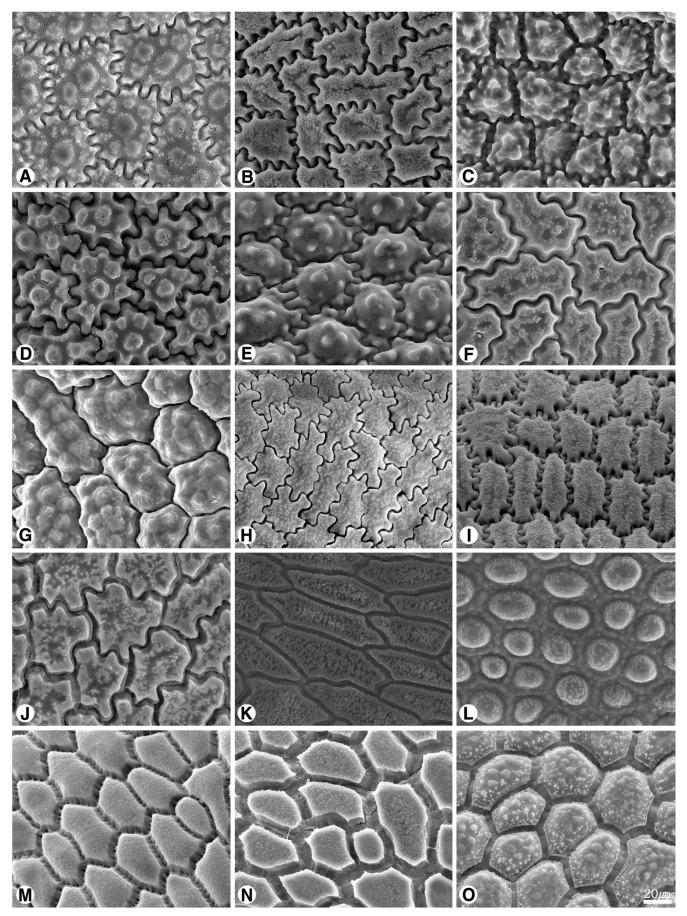
Scanning electron micrographs of seed testa in *Allium*. (**A**–**I**) subg. *Allium*: (**A**) *A*. *filidens* (sect. *Allium*); (**B**) *A. caesium* “101a”; (**C**) *A*. *caesium* “101b”; (**D**) *A*. *caeruleum*; (**E**) *A*. *svetlanae*; (**F**) *A*. *tatyanae* (sect. *Caerulea*); (**G**) *A*. *turkestanicum* (sect. *Mediasia*); (**H**) *A*. *anisotepalum* (sect. *Minuta*); (**I**) *A*. *pallasii* (sect. *Pallasia*); (**J**,**K**) subg. *Butomissa*: (**J**) *A*. *oeroprasum* (sect. *Austromotana*); (**K**) *A*. *ramosum* (sect. *Butomissa*); (**L**–**O**) subg. *Cepa*: (**L**) *A*. *fedschenkoanum* (sect. *Annuloprason*); (**M**) *A*. *altaicum*; (**N**) *A*. *galanthum*; (**O**) *A*. *oschaninii* (sect. *Cepa*).

**Figure 6:**
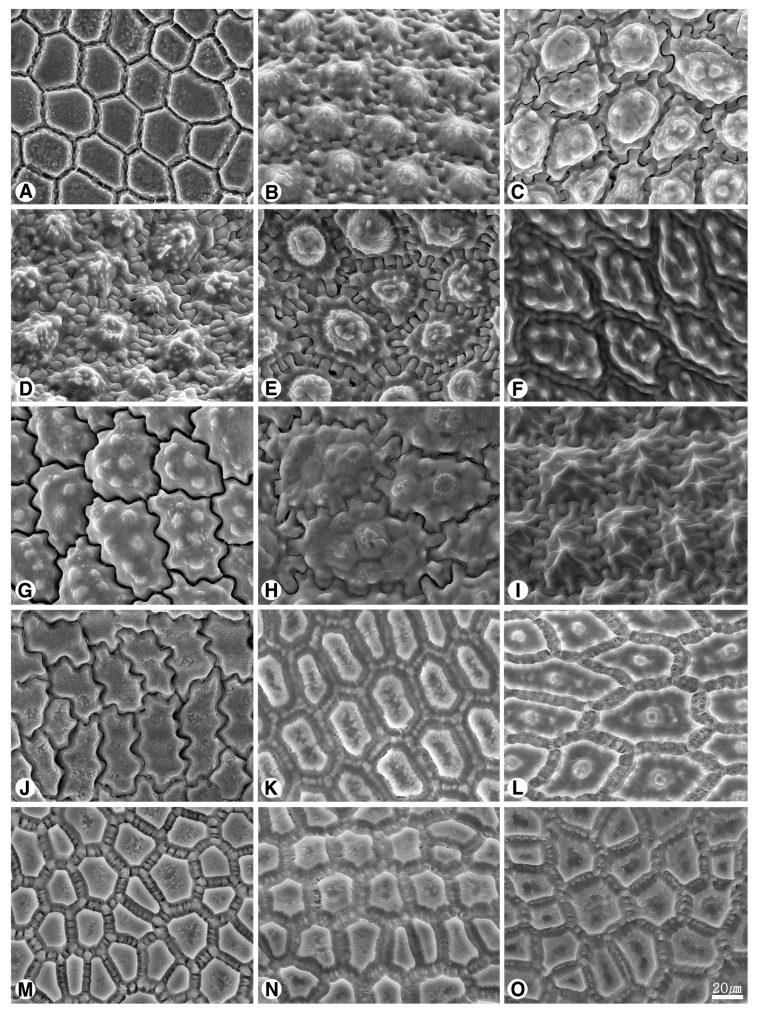
Scanning electron micrographs of seed testa in *Allium*. (**A**) *A*. *maximowiczii*, (sect. *Schoenoprasum*, subg. *Cepa*); (**B**–**J**) subg. *Melanocrommyum*: (**B**) *A*. *saposhnikovii* (sect. *Acmopetala*); (**C**) *A*. *alexianum*; (**D**) *A*. *caspium*; (**E**) *A*. *protensum* (sect. *Kaloprason*); (**F**) *A*. *karataviense* (sect. *Miniprason*); (**G**) *A*. *altissimum*; (**H**) *A*. *stipitatum* (sect. *Procerallium*); (**I**) *A*. *taeniopetalum* (sect. *Stellata*); (**J**) *A*. *viridiflorum* (sect. *Verticillata*); (**K**–**O**) subg. *Polyprason*: (**K**) *A*. *carolinianum*; (**L**) *A*. *hymenorrhizum*; (**M**) *A*. *platyspathum* subsp. *amblyophyllum*; (**N**) *A*. *platyspathum* subsp. *amblyophyllum*; (**O**) *A*. *platyspathum* subsp. *platyspathum* (sect. *Falcatifolia*).

**Figure 7:**
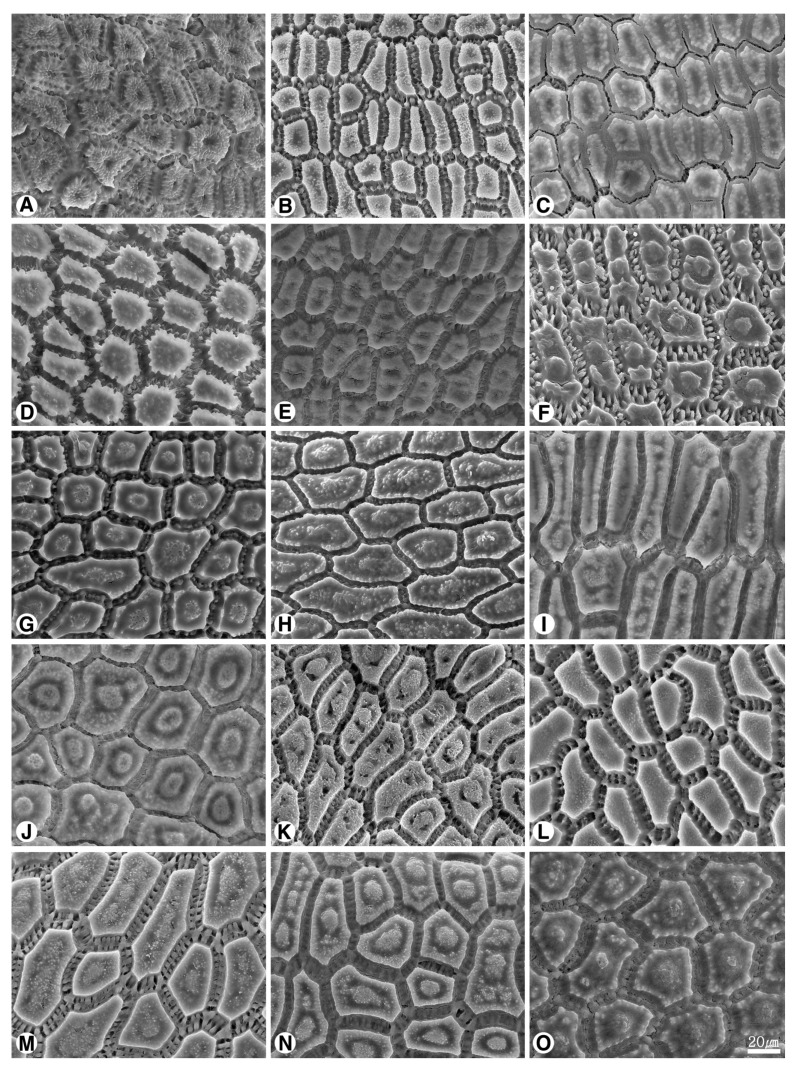
Scanning electron micrographs of seed testa in *Allium*. (**A**–**E**) subg. *Polyprason*: (**A**) *A. korolkowii* (sect. *Falcatifolia*); (**B**) *A. kirilovii*; (**C**) *A. obliquum*; (**D**) *A. petraeum*; (**E**) *A. tianschanicum* (sect. *Oreiprason*); (**F**–**O**) subg. *Reticulatobulbosa*: (**F**) *A. barsczewskii*; (**G**) *A. dolichostylum*; (**H**) *A. jodanthum* (sect. *Campanulata*); (**I**) *A. amphibolum* “136a”; (**J**) *A. amphibolum* “136b”; (**K**) *A. clathratum*; (**L**) *A. leucocephalum*; (**M**) *A. malyschevii*; (**N**) *A. strictum* (sect. *Reticulatobulbosa*); (**O**) *A. trachyscordum* (sect. *Scabriscapa*).

**Figure 8:**
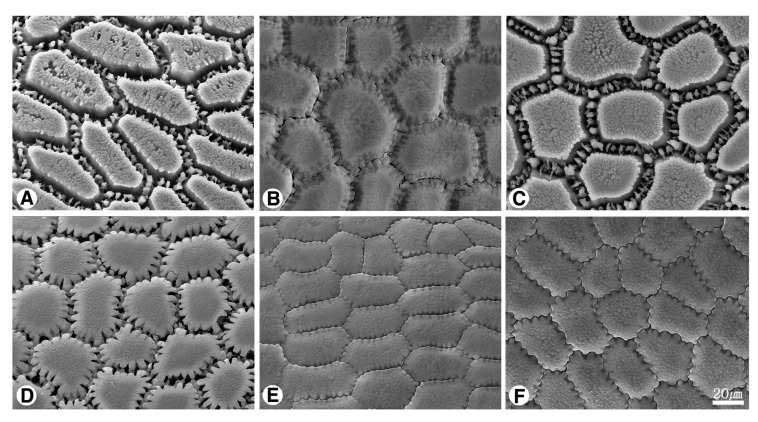
Scanning electron micrographs of seed testa in *Allium*. (**A**–**F**) subg. *Rhizirideum*: (**A**) *A. bidentatum*; (**B**) *A. polyrhizum* (sect. *Caespitosoprason*); (**C**) *A. austrosibiricum* (sect. *Rhizirideum*); (**D**) *A. anisopodium*; (**E**) *A. tenuissimum*; (**F**) *A. vodopjanovae* (sect. *Tenuissima*).

*Allium* seeds display a high degree of variation with potential taxonomic value. The epidermal cells of the seed coat, rather than tightly fitting together, form small voids or channels between them. *Allium* testa topography usually consists of anticlinal cell walls, undulation pattern, and micro-relief of the periclinal cell walls, sometimes divided into a central and a peripheric anticlinal field (Table S1; Figure 4, Figure 5, Figure 6, Figure 7 and Figure 8). Our study indicates that the moderately flat to convex periclinal walls of the *Allium* seed coat tend to have various-sized verrucae or granules in all investigated taxa. The types of anticlinal cell boundaries in *Allium* seed coats are straight, irregularly-curved or variously undulated with different amplitudes and wavelengths (Figure 4), and the shapes of undulations were categorized as U-, S-, or Omega-type. Some species also exhibit a well-developed anticlinal layer of unknown origin, perhaps cuticle, waxy material, or mucopolysaccharides secreted by the seed coat (Figure 6K–O, Figure 7A–O). This matter creates striation or ribbed patterns varying from obscure to prominent, making the observation of the actual boundaries difficult.

#### 2.2.1. Subgenus *Allium*

Eight species and nine accessions (*A. filidens*, *A. caesium*, *A. caeruleum*, *A. svetlanae*, *A. tatyanae*, *A. turkestanicum*, *A. anisotepalum*, and *A. pallasii*), belonging to five sections (*Allium*, *Caerulea*, *Mediastia*, *Minuta* and *Pallasia*), were investigated (Table S1, Figure 1A–I, Figure 5A–I). The seed shapes were oval-flattened (Figure 1A), elliptical-angular (Figure 1B), oval-angular (Figure 1C,D,F,I), oval-spherical (Figure 1E,G), or elliptical-flattened (Figure 1H). The size of seeds ranged between 2.04–3.49 mm in length and 1.06–2.94 mm in width. The L/W ratio ranged between 1.2–2.08.

The seed testa sculptures were showing substantial variations in the studied species. The anticlinal walls were U-type in *A. filidens*, *A. caesium* “101a”, *A. svetlanae* and *A. pallasii* (Figure 5A,B,E,I), U- to Omega-type in *A. caesium* “101b” and *A. caeruleum* (Figure 5C,D), S-type in *A. turkestanicum* (Figure 5G), or irregularly-curved in *A. tatyanae* and *A. anisotepalum* (Figure 5F,H). The periclinal walls were more or less convex with large verrucae or densely granules in *A. filidens*, *A. caesium* “101b”, *A. caeruleum*, *A. svetlanae*, *A. turkestanicum* and *A. pallasii* (Figure 5A,C–E,G,I), or flat to convex with small verrucae or granules in *A. caesium* “101a”, *A. tatyanae* and *A. anisotepalum* (Figure 5B,F,H).

#### 2.2.2. Subgenus *Butomissa (Salisb) N. Friesen*

Two species (*A. oreoprasum* and *A. ramosum*), belonging to sects. *Austromotana* and *Butomissa* respectively, were studied (Table S1; Figure 1J,K, Figure 5J,K). The seed shapes were oval-angular in *A. oreoprasum* (Figure 1J) and oval-hemispherical in *A. ramosum* (Figure 1K). The size of seeds ranged between 3.42–4.08 mm in length and 2.11–2.71 mm in width. The L/W ratio ranged between 1.27–1.94.

The anticlinal walls were irregularly-curved in both *A. oreoprasum* (Figure 5J) and *A. ramosum* (Figure 5K), but the former showed much sharper and clearer undulation and the latter showed nearly-straight walls. The periclinal walls were more or less flat with many small verrucae with marginal granules in *A. oreoprasum* (Figure 5J), or with densely granules in *A. ramosum* (Figure 5K).

#### 2.2.3. Subgenus *Cepa (Mill.) Radić*

Five species (*A. fedschenkoanum*, *A. altaicum*, *A. galanthum*, *A. oschaninii*, and *A. maximowiczii*), belonging to three sections (*Annuloprason*, *Cepa*, and *Schoenoprasum*), were examined (Table S1; Figure 1L–P, Figure 5L–O, Figure 6A). All the investigated seeds were elliptical-angular (Figure 1L,P) or oval-angular (Figure 1M–O) in shape. The size of seeds ranged between 2.23–4.47 mm in length and 1.12–2.65 mm in width. The L/W ratio ranged between 1.45–2.40.

The seeds in this subgenus showed nearly-straight anticlinal walls (Figure 5L–O, Figure 6A). On the other hand, the periclinal walls presented colliculose cellular center and verrucae in *A. fedschenkoanum* (Figure 5L), or convex verrucae with granules in *A. altaicum*, *A. galanthum*, *A. oschaninii*, and *A. maximowiczii* (Figure 5M–O, Figure 6A).

#### 2.2.4. Subgenus *Melanocrommyum (Webb et Berth.) Rouy*

Nine species (*A. saposhnikovii*, *A. alexeianum*, *A. caspium*, *A. protensum*, *A. karataviense*, *A. altissimum*, *A. stipitatum*, *A. taeniopetalum*, and *A. viridiflorum*), belonging to six sections (*Acmopetala*, *Kaloprason*, *Miniprason*, *Procerallium*, *Stellata*, and *Verticilata*), were investigated (Table S1; Figure 2A–I, Figure 6B–J). All the observed seeds were characterized by oval-angular (Figure 2A), oval-hemispherical (Figure 2B,C,E,G–I), or oval-spherical (Figure 2D,F) based on the shapes. The seed length ranged between 1.89–4.01 mm and the seed width were between 1.12–2.65 mm. The L/W ratio ranged between 1.13–1.52.

The anticlinal walls were characterized by U- to Omega-type in *A. saposhnikovii*, *A. alexeianum*, *A. caspium*, *A. protensum*, *A. stipitatum* and *A. taeniopetalum* (Figure 6B–E,H,I), S- to U- type in *A. karataviense* (Figure 6F), or irregularly-curved in *A. viridiflorum* (Figure 6J). The periclinal walls were flat to convex with verrucae in *A. saposhnikovii*, *A. alexeianum*, *A. caspium*, *A. protensum*, *A. stipitatum*, *A. taeniopetalum*, *A. protensum* and *A. karataviense* (Figure 6B–I), or with densely prominent granules in *A. viridiflorum* (Figure 6J).

#### 2.2.5. Subgenus *Polyprason Radić*

Nine accessions of eight species (*A. carolinianum*, *A. hymenorrhizum*, *A. platyspathum* subsp. *platyspathum*, *A. platyspathum* subsp. *amblyophyllum*, *A. korolkowii*, *A. kirilovii*, *A. obliquum*, *A. petraeum*, and *A. tianschanicum*), belonging to two sections (*Falcatifolia* and *Oreiprason*), were examined (Table S1; Figure 2J–Q, Figure 3A, Figure 6K–O, Figure 7A–E). The investigated seeds were elliptical-angular (Figure 2J,O, Figure 3A), oval-flattened (Figure 2K), or oval-angular (Figure 2L–N,P,Q) by the shapes. The seed length ranged between 2.82–3.91 mm and the seed width ranged between 1.69–2.27 mm. The L/W ratio ranged between 1.44–2.27.

All the investigated seeds showed nearly-straight anticlinal walls (Figure 6L–O, Figure 7A–D). The more or less convex periclinal walls showed one central verruca and marginal small verrucae in *A. hymenorrhizum* (Figure 6L), or densely granules/small verrucae in the others (Figure 6M–O, Figure 7A–D).

#### 2.2.6. Subgenus *Reticulatobulbosa (Kamelin) N. Friesen*

Ten accessions from nine species (*A. barszewskii*, *A. dolychostylum*, *A. jodanthum*, *A. amphibolum*, *A. clathratum*, *A. leucocephalum*, *A. malyschevii*, *A. strictum*, and *A. trachyscordum*), belonging to three sections (*Campanulata*, *Reticulatobulbosa*, and *Scabriscapa*), were investigated (Table S1; Figure 3B–J, Figure 7F–O). All the investigated seeds were characterized by elliptical-flattened (Figure 3B,D), elliptical-angular (Figure 3E–I), oval-flattened (Figure 3C), or oval-angular (Figure 3J) shape. The seed length ranged between 2.72–3.93 mm and the width ranged between 1.30–2.35 mm. The L/W ratio ranged between 1.36–2.31.

The anticlinal walls were nearly-straight in all the observed species (Figure 7F–O). The periclinal walls usually showed various verrucae in most species (Figure 7F–O), but having granules in *A. leucocephalum* (Figure 7L).

#### 2.2.7. Subgenus *Rhizirideum (G.Don. ex Koch) Wendelbo s.s.*

Six species (*A. bidentatum*, *A. polyrhizum*, *A. austrosibiricum*, *A. anisopodium*, *A. tenuissimum*, and *A. vodopjanovae*), belonging to three sections (*Caespitosoprason*, *Rhizirideum*, and *Tenuissima*), were observed (Table S1; Figure 3K–P, Figure 8A–F). All the examined species showed oval-angular (Figure 3K,L,N–P) or oval-hemispherical (Figure 3M) in shape. The seed length ranged between 1.74–2.94 mm and the width ranged between 1.30–2.35 mm. The L/W ratio ranged between 1.31–1.77.

The anticlinal walls were distinguished by nearly-straight (Figure 8A–C) or S-type (Figure 8D–F). The periclinal wall was flat to more or less convex with densely granules in all observed samples (Figure 8A–F).

## 3. Discussion

Worldwide, more than 460 taxa of *Allium* have been investigated for their seed macro- and micro-morphological characteristics [14,15,16,17,18,19,20,21,23,24,25,26,27,28,29,30,31,32,33,43,44,45,46,47,48,49]. The highest number of species have been studied from Turkey, China (Xinjiang), and Iran with 62, 38 and 23 species, respectively [28,31,32]. The results of this study agree with the former investigations on seed morphology of the genus with respect to their shape, size, and surface sculpture (e.g., Celep et al. [28]; Duman et al. [29]; Choi et al. [30]; Lin and Tan [31]; Veiskarami et al. [32]). In particular, Choi et al. [30], Celep et al. [28] and Veiskarami et al. [32] carried combined-investigation on both seed morphology and seed surface microstructure in the genus *Allium*. In this research, we observed 48 species and 51 taxa of *Allium* collected from Mongolia, Uzbekistan, and Kyrgyzstan, 18 species of which were firstly studied.

**Figure 9:**
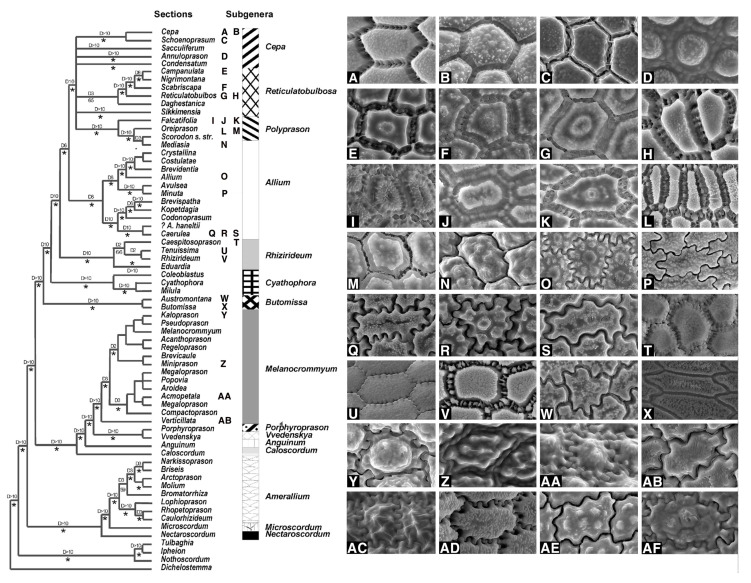
Seed testa characteristics of selected sections in *Allium* phylogeny tree (modified from Friesen et al., 2006). (**A**–**D**) subg. *Cepa*: (**A**) *A. altaicum*; (**B**) *A. oschaninii*; (**C**) *A. maximowiczii*; (**D**) *A. fedschenkoanum* *; (**E**–**H**) subg. *Reticulatobulbosa*: (**E**) *A*. *dolichostylum*; (**F**) *A. trachyscordum*; (**G**) *A. amphibolum*; (**H**) *A. leucocephalum*; (**I**–**M**) subg. *Polyprason*: (**I**) *A. korolkowii*; (**J**) *A. carolinianum* *; (**K**) *A. hymenorrhizum*; (**L**) *A. kirilovii*; (**M**) *A. obliquum*; (**N**–**S**,**AD**) subg. *Allium*: (**N**) *A. turkestanicum* *; (**O**) *A. filidens*; (**P**) *A*. *anisotepalum*; (**Q**) *A. caesium*; (**R**) *A. svetlanae*; (**S**) *A. tatyanae*; (**T**–**V**) subg. *Rhizirideum*: (**T**) *A. polyrhizum* *; (**U**) *A. anisopodium*; (**V**) *A*. *austrosibiricum*; (**W**–**X**) subg. *Butomissa*: (**W**) *A*. *oreoprasum* *; (**X**) *A*. *ramosum* *; (**Y**–**AC**,**AE**,**AF**) subg. *Melanocrommyum*: (**Y**) *A. alexeianum*; (**Z**) *A. karataviense* *; (**AA**) *A*. *saposhnikovii*; (**AB**) *A. viridiflorum*; (**AC**) *A. taeniopetalum* (sect. *Stellata*); (**AD**) *A. pallasii* * (sect. *Pallasia*); (**AE**) *A. altissimum*; (**AF**) *A. stipitatum* (sect. *Procerallium*). Figure (**AC**–**AF**) were not included in Friesen et al. (2006). (The type species of section marked by asterisk).

Our results showed that the straight anticlinal walls were dominant in subg. *Cepa*, *Reticulatobulbosa* and *Polyprason*, and the periclinal walls were distinguished by central big verrucae and dense granules (Figure 9A–M). The U-, Omega- and S-type undulated anticlinal walls and convex periclinal walls with several large verrucae or marginal verrucae were dominant in subg. *Allium* and *Melanocrommyum* (Figure 9N–S,Y–AF). The subg. *Butomissa* differed by having irregularly-curved or nearly-straight anticlinal walls (Figure 9W,X). More variable anticlinal walls were found in subg. *Rhizirideum* to follow straight, S- to straight and S-type (Figure 9T–V). The shape and size of seeds can be considered as taxonomically important characteristics in the species and section levels of the genus *Allium* [28,29,32]. Based on our results, the seed shape and size was substantially diverse (Table S1; Figure 1, Figure 2 and Figure 3). Especially, the elliptical seeds were found in subg. *Reticulatobulbosa*, as followed by oval-hemispherical or oval-spherical seeds in subg. *Melanocrommyum*, or oval-angular seeds in subg. *Cepa*, *Polyprason* and *Rhizirideum*. On the other hand, seed shape and size were quite diverse in subg. *Allium*.

Consequently, we conclude that seed characteristics such as size, shape, and seed testa features show significant variability, providing key characteristics to support taxonomic relationships and major clades revealed in the molecular phylogeny of the genus *Allium* (Figure 9).

### 3.1. Subgenus Allium

The subg. *Allium* is a large and monophyletic group with more than 375 species and 35 subspecies divided into 18 sections [2,3,49]. However, a detailed phylogenetic classification of this taxonomically complex subgenus is still missing [49]. Currently, about 140 taxa were investigated by seed morphological studies from different countries. The most studied species were from sects. *Allium* and *Codonoprasum* in this subgenus. Seed shape, size, and seed testa structures are highly variable; for example, the anticlinal walls have U-, Omega- or S-type undulation, or straight in the species and sections.

Section *Allium*: Only *A. filidens* collected form Uzbekistan has been investigated for the first time in this section. Previous studies suggested that the sect. *Allium* had convex periclinal walls with several verrucae, primarily one large verruca in the center, and strongly U- to S-type anticlinal walls [14,16,17,18,19,20,24,32]. Our results agreed with the former studies on anticlinal and periclinal walls structure (Figure 5A). Moreover, seed size and shape were considerably variable in the species of the section [28].

Section *Caerulea* (Omelcz.) F.O. Khassanov: Four species (*A. caesium*, *A. caeruleum*, *A. svetlanae* and *A. tatyanae*) collected from Uzbekistan were investigated in this section. Among them, *A. svetlanae* and *A. tatyanae* were firstly described. According to the previous studies, the species of this section has convex periclinal walls with verrucae and densely granules whereas the anticlinal walls vary with the combined U- to S-type undulations, U- to Omega-type undulations, or straight walls in *A. caesium*, *A. caeruleum, A. capitellaum*, and *A. sairamense* [30,31,32]. Lin and Tan [31] observed only S-type anticlinal walls for *A. caesium* and *A. caeruleum* from China (Xinjiang). However, we found combined U- and Omega-type anticlinal walls in both species (Figure 5B–D). Our results agreed with Choi et al. [30] who described *A. caeruleum* as having U- to Omega-type of anticlinal wall.

Section *Mediasia* F.O. Khassanov et S.C. Yengalycheva et N. Friesen: *A. turkestanicum*, the type species of the section [2] collected from Uzbekistan was investigated for the first time. This species differs from other sections in subg. *Allium* by having the anticlinal walls with S-type undulation (Figure 5G). The seed shape (oval-spherical, Figure 1E) resembled the *A. svetlanae* (sect. *Caerulea*) in terms of having slightly larger seeds than this species. Our results showed that seed testa, seed shape, and size of *A. turkestanicum* (sect. *Mediasia*) strongly support the phylogenetic results of Friesen et al. [2].

Section *Minuta* F.O. Khassanov: Only *A. anisotepalum* was firstly examined from Uzbekistan in this section. The irregularly-curved anticlinal walls of this species (Figure 5H) differed from other sections in the subg. *Allium*, except *A*. *tatyanae* (sect. *Allium*, subg. *Allium*). The anticlinal and periclinal walls of *A. anisotepalum* were very similar to those of *A. tatyanae*, but seed shape and size were different from each other.

Section *Pallasia* (Tzagolova.) F.O. Khassanov, R.M. Fritsch et N. Friesen: Only *A. pallasii* collected from Kyrgyzstan was studied. Our results were congruent with those of Kruse [20] and Lin and Tan [31] as this species is characterized by U-type anticlinal walls, and convex periclinal walls with dense granulation (Figure 5I). The seed size and shape were observed for the first time in this study.

### 3.2. Subgenus Butomissa (Salisb.) N. Friesen

Section *Austromontana* N. Friesen and section *Butomissa*: Two species, *A. oreoprasum* (sect. *Austromontana*) and *A. ramosum* (sect. *Butomissa*), from Kyrgyzstan and Mongolia, respectively, were investigated. Both species were the type species of each section. This subgenus has two sections (*Austromontana* and *Butomissa*) and four species that are usually placed in the third evolutionary line [2,3]. Previous studies suggested that nearly-straight anticlinal walls and granulose sculptures on the periclinal walls were the characteristic features of this subgenus [17,18,20,23]. Additionally, Lin and Tan [31] recognized arched to S-type anticlinal walls and periclinal walls with intermediate verrucae in *A. oreoprasum* from China. From our results, we confirmed irregularly-curved anticlinal walls and periclinal walls with many verrucae in *A. oreoprasum* (Figure 5J). Choi et al. [30] described irregularly-curved anticlinal wall boundaries covered with granulate periclinal walls in two Chinese species: *A. ramosum* and *A. tuberosum* (sect. *Butomissa*). We also observed irregularly-curved anticlinal walls (Figure 5K) in *A. ramosum* from Mongolia. The shape of seeds was easily distinguished within two sections by its oval-angular shape in *A. oreoprasum* (Figure 1J) and oval-hemispherical shape in *A. ramosum* (Figure 1K). Our results concurred with Friesen et al. [2] and Li et al. [3], and their phylogenetic results.

### 3.3. Subgenus Cepa (Mill.) Radić

The seed testa sculpture of most species representing the subg. *Cepa* had densely granulate periclinal walls and straight to arched anticlinal walls [19,20,27,30,31,32]. Our results indicated the seed shape and size were distinguished from each section by oval-angular seeds in sect. *Cepa* and elliptical-angular seeds in sects. *Annuloprason* and *Schoenoprasum*.

Section *Annuloprason* T.V. Egorova: Only *A. fedschenkoanum* sampled from Kyrgyzstan was firstly described. Lin and Tan [31] observed S-type anticlinal walls and periclinal walls with tuberculate or many intermediate verrucae for two species from China (Xinjiang). We observed straight anticlinal walls and periclinal walls with colliculose cellular center and verrucae (Figure 5L), which is very similar to that of *A. astrosanguineum* [31]. Generally, those species were distinguished by only their inflorescence color and habitat.

Section *Cepa*: Three species (*A. altaicum, A. galanthum* and *A. oschaninii*) from Mongolia and Kyrgyzstan were observed in this section. Among them, seed testa sculpture of *Allium altaicum* was firstly investigated. Lin and Tan [31] reported straight to arched anticlinal walls and the periclinal walls with intermediate verrucae for *A. galanthum* from China (Xinjiang). In contrast, we found straight anticlinal walls and densely granulate periclinal walls (Figure 5N) for *A. galanthum* from Mongolia. Our observations agreed with the results of Veiskarami et al. [32] because *A. oschaninii* had straight anticlinal walls and periclinal walls with large verrucae (Figure 5O). Although Veiskarami et al. [32] discovered a broadly ellipsoid-ovoid seed for *A. oschaninii* from Iran, we found oval-angular seed for the same species from Kyrgyzstan (Figure 1O).

Section *Schoenoprasum* Dumort.: Only *A. maximowiczii* was studied from Mongolia. The straight anticlinal walls and the periclinal walls with intermediate verrucae or densely granules are dominant in this section [16,30,31]. Our results for *A. maximowiczii* were also consistent with the former studies. The elliptical-angular seeds were found in *A. maximowiczii* and *A. schoenoprasum* from China and Canada, respectively [25,30]. We also discovered similar seed shapes for *A. maximowiczii* (Figure 1P).

### 3.4. Subgenus Melanocrommyum (Webb et Berth.) Rouy

This subgenus consists of 140 species and it is the second largest subgenus of the second evolutionary line in genus *Allium* [7,23]. Seed testa structure of 120 taxa has been studied in this subgenus [16,17,18,19,20,23,24,28,31,33,45,46,48]. The subg. *Melanocrommyum* was characterized by convex periclinal walls with several large verrucate sculptures and combined S- to Omega-type undulated anticlinal walls [23,28]. Our results described the seed of this subgenus as generally well-distinguished from other species of the genus *Allium* by its oval-spherical or oval-hemispherical shapes. However, the seed size was more variable with respect to the studied species.

Section *Acmopetala* R.M. Fritsch: Only *A. saposhnikovii* from Kyrgyzstan was examined in this section. Fritsch et al. [23] found anticlinal walls of moderate wavelength with high amplitude and verrucate periclinal walls for this species. We found one central verrucate periclinal walls and U-type anticlinal walls (Figure 6B). The seed size and shape was firstly described.

Section *Kaloprason* C. Koch: Three species of *A. alexeianum*, *A. caspium*, and *A. protensum* were studied from Uzbekistan in this section. Fritsch et al. [23] and Neshati and Fritsch [24] observed the combined U- to Omega-type anticlinal walls and convex periclinal walls with verrucae and granules for the several species from Iran. Our results matched with their findings (Figure 6C–E). We also firstly observed the seed size and shape.

Section *Miniprason* R.M. Fritsch: The single species, *A. karataviense* from Uzbekistan, was investigated in this section. Bednorz et al. [27] found roundish seeds (3.1 × 2.9 mm) for *A. karataviense* from Poland. However, we recognized that the seeds were oval-semicircular and larger (4.36 × 3.44 mm) for the same species. On the other hand, Kruse [20] and Fritsch et al. [23] investigated anticlinal walls of Omega-type undulations with low amplitude in *A. karataviense* from Middle Asia, but our results re-confirmed the result of Bednorz et al. [27] for S- to U-type undulated anticlinal walls and the periclinal walls with 4–10 prominent verrucae (Figure 6F).

Section *Procerallium* R.M Fritsch: According to Fritsch and Abbasi [7], this section is divided into two subsections: subsect. *Elatae* includes two species (*A. altissimum* and *A. stipitatum*) and subsect. *Costatae* includes six species. We investigated *A. altissimum* and *A. stipitatum* from Uzbekistan in this section. We found S-type anticlinal wall and several big verrucate periclinal wall for *A. altissimum* (Figure 6G), quite similar to the results of Fritsch et al. [23] and Neshati and Fritsch [24]. Kruse [19] reported straight anticlinal wall for *A. stipitatum* but we observed U- to Omega-type anticlinal wall for same species (Figure 6H). Neshati and Fritsch [24] examined seed shape and size (length x width) for *A. altissimum* and *A. stipitatum* (broadly ovate, 4 × 3 mm and long ovate, 4 × 2.5 mm, respectively) from Iran. Our examined seeds of both species were smaller (oval-spherical, 2.98 × 2.65 mm and oval-hemispherical, 3.84 × 2.96 mm, respectively) than the results of Neshati and Fritsch [24].

Section *Stellata* (F.O. Khass. and R.M. Fritsch) R.M Fritsch: *Allium taeniopetalum* from Uzbekistan was studied for the first time. The seed coat characteristics of this species were very similar to sect. *Acmopetala* by having U- to Omega-type anticlinal walls (Figure 6I). The seed shape of *A. taeniopetalum* was also similar to *A. virdiflorum* (sect. *Verticllata*) but the seed was notably smaller than the latter.

Section *Verticillata* Kamelin: The single species of *A. viridiflorum* was studied from Uzbekistan in this section. Fritsch et al. [23] found shallowly undulated anticlinal walls and finely granulate periclinal walls for this species from the type location. We found irregularly-curved anticlinal walls (Figure 6J) and quite similar to that of the former results by Fritsch et al. [23]. The seed testa of *A. viridiflorum* is similar to *A. tatyanae* (sect. *Allium*), *A. anisotepalum* (sect. *Minuta*, subg. *Allium*), and *A. oreoprasum* (sect. *Austromotana*, subg. *Butomissa*). However, their seed shapes are different from each other. The seed size and shape of *A. viridiflorum* was firstly observed.

### 3.5. Subgenus Polyprason Radić

In this subgenus, several species of sects. *Falcatifolia* and *Oreiprason* were studied by Lin and Tan [31]. Our results agreed with the findings of Lin and Tan [31], i.e., these two sections have very similar seed characteristics. Our results showed that all the studied species had almost same characteristics in terms of their straight anticlinal walls and densely granulate periclinal walls except *A. hymenorrhizum* having one central verruca and small marginal verrucae. For these sections, the seed shape was mostly oval-angular or elliptical-angular except *A. hymenorrhizum* with oval-flattened. Based on our results, seed shape and seed testa pattern of species in subg. *Polyprason* were similar to the species of subg. *Reticulatobulbosa*.

Section *Falcatifolia* N. Friesen: Four species of *A. carolinianum*, *A. korolkowii*, *A. hymenorrhizum*, *A. platyspathum* subsp. *platyspathum*, and *A. platyspathum* subsp. *amblyophyllum* were studied from Kyrgyzstan, Uzbekistan, and Mongolia. Lin and Tan [31] presented straight to arched anticlinal walls and convex periclinal walls with intermediate verrucae for the above-mentioned species. Our results showed similar findings to those of Lin and Tan [31] for the anticlinal walls. However, we found that the periclinal walls were indistinctly granulate expect *A. hymenorrhizum*. Particularly, the seed shape and the testa sculpture of *A. hymenorrhizum* were unexpectedly variable in the same taxa from different countries [18,28,43]. For example, Filimonova [43] and Celep et al. [28] reported oblong and narrowly ovate seeds for *A. hymenorrhizum* from central Asia and Iran, respectively; however, we cannot confirm their results because we observed the oval-flattened shape (Figure 2K). Lin and Tan [31] observed straight anticlinal wall and intermediate verrucae from China (Xinjiang). Our findings were similar to the results of Kruse [18] and Celep et al. [28] for straight anticlinal wall and central verrucae with marginal granules. These differences are likely to be caused by sampling related but not-identical taxa. Currently, *A. hymenorrhizum* is not a clearly recognized taxon, and thus, further research is required in the future. Lin and Tan [31] treated *A. korolkowii* as a member of subg. *Reticulatobulbosa*. However, our results suggested that the seed shape and the testa sculpture of *A. korolkowii* were similar to the species of sect. *Falcatifolia* of subg. *Polyprason*, not to the subg. *Reticulatobulbosa*. In addition, *A. korolkowii* has different bulb tunic characteristics from subg. *Reticulatobulbosa*. Therefore, we treated *A. korolkowii* as a member of sect. *Falcatifolia* of subg. *Polyprason* in this study.

Section *Oreiprason* F. Herm: We investigated four species (*A. kirilovii*, *A. obliquum*, *A. petraeum*, and *A. tianschanicum*) from Kyrgyzstan in this section. Seed testa sculpture of *A. kirilovii* (newly described by Seregin et al. [11]) and *A. petraeum* were firstly reported. In addition, eight species of this section were studied from China (Xinjiang) by Lin and Tan [31], and all of them had straight to arched anticlinal walls and periclinal walls with intermediate verrucae or many granules. Our results also showed similar findings to those of Lin and Tan [31]. The seed sizes and shapes of all the species were firstly observed in this study.

### 3.6. Subgenus Reticulatobulbosa (Kamelin) N. Friesen

This subgenus comprises 80 species belonging to seven sections. It is placed at the third evolutionary line in genus *Allium* [2,3]. Friesen et al. [2] described three new sections (*Scabriscapa*, *Nigrimontana*, and *Sikkimensia*) based on the phylogenetic result. Seed testa sculptures of most species in this subgenus were investigated from sects. *Campanulata* and *Reticulatobulbosa*. The straight anticlinal walls and one or several large verrucae with granules of the periclinal walls were dominant [17,19,24,30,31]. Our findings also clearly supported the previous results for the subg. *Reticulatobulbosa*. We found elliptical-angular or elliptical-flattened shape of seeds in sects. *Campanulata* and *Reticulatobulbosa*. *Allium trachyscordum* (sect. *Scabriscapa*) was distinguished from the other sections by its oval-angular seed shape. The seed shape of all species had low variations for each species in this subgenus and it is easily recognized from other subgenera except some species of subg. *Polyprason*.

Section *Campanulata* N. Friesen: Three species of *A. barszewskii*, *A. dolychostylum*, and *A. jodanthum* were studied from Uzbekistan and Kyrgyzstan. Seed testa sculptures of *A. dolychostylum* and *A. jodanthum* were firstly investigated. Kruse [19] observed U-type undulated anticlinal walls with verrucate periclinal walls for *A. barszweskii*. However, our results did not confirm that of Kruse [19]. Instead, we found similar results to Neshati and Fritsch [24]’s straight anticlinal walls and verrucate periclinal walls (Figure 7F–H).

Section *Reticulatobulbosa* s.s.: We investigated five species—*A. amphibolum*. *A. clathratum*, *A. leucocephalum*, *A. malyschevii*, and *A. strictum*—from Mongolia for the first time in this section except *A. strictum*. Choi et al. [30] found verrucate periclinal walls in *A. koreanum* and *A. splendens* from Korea and China, respectively. Lin and Tan [31] described periclinal walls with large central verrucae for *A. strictum* and *A. flavidum* from China (Xinjiang). Our results showed that periclinal walls had one central verruca with marginal verrucae, or had dense granulation. Choi et al. [30] and Lin and Tan [31] also reported that straight to arched anticlinal walls in this section; our results confirmed their findings. Within this section, seed sizes were quite variable but seed shapes were elliptical-angular in all the studied species.

Section *Scabriscapa* (Tscholok.) N. Friesen: The single species of *A. trachyscordum* from Kyrgyzstan was firstly investigated. Neshati and Fritsch [24] reported convex periclinal walls with central verrucae and strip like anticlinal walls for *A. scabriscapum* from Iran. Our studied species, *A. trachyscordum*, had periclinal walls with one central verruca and marginal small verrucae, and straight anticlinal walls (Figure 7O). The seed shape of this species was oval-angular (Figure 3J), easily distinguished from other species of the sect. *Reticulatobulbosa*.

### 3.7. Subgenus Rhizirideum (G. Don ex Koch) Wendelbo s.s.

Section *Caespitosoprason* N. Friesen: Recently, Friesen et al. [13] proposed that sect. *Caespitosoprason* includes eight species in central Asia and it is a synonym of sect. *Rhizomatosa* based on the molecular phylogeny. We studied two species of *A. bidentatum* and *A. polyrhizum* (the type species of sect. *Caespitosoprason*) from Mongolia for this section. Straight anticlinal walls and convex periclinal walls with intermediate verrucae or granules were dominant in this section [19,30,31], which is supported by our results as well. In addition, Lin and Tan [31] reported the same results for *A. caespitosum* (the type species of sect. *Rhizomatosa*) from China (Xinjiang). These results confirmed the recent phylogenetic work of Friesen et al. [13].

Section *Rhizirideum* s.s: This section is a strongly monophyletic group including 24 species. It is distributed mostly in the steppe areas of the Eurasian temperate zone [2,12]. We investigated only *A. austrosibiricum* from Mongolia for the first time. Choi et al. [30] described an oval-hemisphere seed shape for several species from China and Korea. Bednorz et al. [27] reported a roundish, elliptical seed shape for *A. nutans* from Poland. Our studied species, *A. austrosibiricum*, from Mongolia also had oval-hemispherical seed shape (Figure 3M). Straight anticlinal walls and many granulate periclinal walls were dominant in this section [27,30,31] and our results for *A. austrosibiricum* were similar to those former results.

Section *Tenuissima* (Tzagolova) Hanelt: The three species of *A. anisopodium*, *A. tenuissimum*, and *A. vodopjanovae* were collected from Mongolia in this section. Seed testa characteristics of *A. vodopjanovae* were firstly described in this study. Kruse [19] found S-type anticlinal walls for *A. anisopodium* and *A. tenuissimum*. Choi et al. [30] also reported S-type anticlinal walls and granulate periclinal walls for *A. anisopodium* and *A. tenuissimum* from China and Korea, respectively. Our results confirmed the findings of Kruse [19] and Choi et al. [30]. The seed shapes were oval-angular, similar to the results of Choi et al. [30].

## 4. Materials and Methods

### 4.1. Taxon Sampling

A total of 51 accessions of 48 species (including two subspecies), belonging to 24 sections of seven subgenera [2,3], were collected between 2014 to 2018 from the central Asian regions. Among them, 19 accessions were from Uzbekistan and 16 each were from Mongolia and Kyrgyzstan. Collection and voucher information is presented in Table S2. Mature seeds were removed from fruits in field, and fixed using 70% ethanol solution.

### 4.2. Light Microscopic Analysis

Seed size and shape were measured and observed under stereomicroscope. Length and width from minimum 20 (to 30) seeds were measured in each accession using the Olympus SZ61 stereoscope (Olympus Co., Tokyo, Japan) with a TrueChrome II camera (Tucsen Photonics Co., Ltd., Fuzhou, China).

### 4.3. Scanning Electron Microscopic Analysis

No special pre-treatments were applied for the preparation of scanning electron microscopy (SEM). Seeds were immersed in absolute ethanol and sputtered with a gold coating in a KIC-IA COXEM Ion-Coater (COXEM Co., Ltd., Daejeon, Korea). In all cases, the seeds of at least five samples per accession were analyzed, characterized, and photographed with a COXEM EM-30 scanning electron microscope (COXEM Co., Ltd., Daejeon, Korea) at 20 kV at the Seed Testing Laboratory of the Baekudaegan National Arboretum, South Korea.

Terminologies for the description of macro- and micro-characteristics are according to Barthlott [50], Kruse [17], Koch et al. [51], Celep et al. [28], and Choi et al. [30].

## 5. Conclusions

We studied seed macro- and micro-morphological characteristics of 48 *Allium* species from Uzbekistan, Kyrgyzstan, and Mongolia. Among them, seed testa features of 18 species were firstly described. Based on our results, we conclude that seed characteristics such as size, shape, and seed testa features showed the significant variability, presenting key characteristics to support taxonomic relationships and major clades exposed in the molecular phylogeny of the genus *Allium*. Most of our results confirmed the previous findings in terms of assuring group-specific patterns for all the sections and subgenera. Particularly, the anticlinal wall characteristics were highly variable and decisive at both the section and species levels. In addition, we discovered the widely varied shape and size of the seeds were key to distinguishing the species level in *Allium*.

## Data Availability

All data generated or analyzed during this study are included in this published article and its supplementary information file.

## Supplementary Materials

Supplementary materials can be found at https://www.mdpi.com/2223-7747/9/9/1239/s1. Table S1. Macro- and micro-morphological characteristics of seed in *Allium* species investigated. Table S2. Voucher specimen information of *Allium* species investigated.

## References

[1] Herden T., Hanelt P., Friesen N. (2016). Phylogeny of *Allium* L. subgenus *Anguinum* (G. Don. ex W.D.J. Koch) N. Friesen (Amaryllidaceae). Mol. Phylogen. Evol..

[2] Friesen N., Fritsch R.M., Blattner F.R. (2006). Phylogeny and new intrageneric classification of *Allium* (Alliaceae) based on nuclear ribosomal DNA its sequences. Aliso.

[3] Li Q.Q., Zhou S.D., He X.J., Yu Y., Zhang Y.C., Wei X.Q. (2010). Phylogeny and biogeography of *Allium* (Amaryllidaceae: Allieae) based on nuclear ribosomal internal transcribed spacer and chloroplast *rps16* sequences, focusing on the inclusion of species endemic to China. Ann. Bot..

[4] Gurushidze M., Mashayekhi S., Blattner F.R., Friesen N., Fritsch R.M. (2007). Phylogenetic relationships of wild and cultivated species of *Allium* section *Cepa* inferred by nuclear rDNA ITS sequence analysis. Plant Syst. Evol..

[5] Gurushidze M., Fritsch R.M., Blattner F.R. (2008). Phylogenetic analysis of *Allium* subg. *Melanocrommyum* infers cryptic species and demands a new sectional classification. Mol. Phylogen. Evol..

[6] Gurushidze M., Fritsch R.M., Blattner F.R. (2010). Species level phylogeny of *Allium* subgenus *Melanocrommyum*: Incomplete lineage sorting, hybridization and *trn*F gene duplication. Taxon.

[7] Fritsch R.M., Abbasi M. (2013). A Taxonomic Review of Allium Subg. Melanocrommyum in Iran.

[8] Wheeler E.J., Mashayekhi S., Mcneal D.W., Columbus J.T., Pires J.C. (2013). Molecular systematics of *Allium* subgenus *Amerallium* (Amaryllidaceae) in North America. Am. J. Bot..

[9] Mashayeckhi S., Columbus J.T. (2014). Evolution of leaf blade anatomy in *Allium* (Amaryllidaceae) subgenus *Amerallium* with a focus on the North American species. Am. J. Bot..

[10] Li M.J., Tan J.B., Xie D.F., Huang D.Q., Gao Y.D., He X.J. (2016). Revisiting the evolutionary events in *Allium* subgenus *Cyathophora* (Amaryllidaceae): Insights into the effect of the Hengduan Mountains Region (HMR) uplift and Quaternary climatic fluctuations to the environmental changes in the Qinghai-Tibet Plateau. Mol. Phylogen. Evol..

[11] Seregin A.P., Anaćkov G., Friesen N. (2015). Molecular and morphological revision of the *Allium saxatile* group (Amaryllidaceae): Geographical isolation as the driving force of underestimated speciation. Bot. J. Linn. Soc..

[12] Sinitsyna T.A., Herden T., Friesen N. (2016). Dated phylogeny and biogeography of the Eurasian *Allium* section *Rhizirideum* (Amaryllidaceae). Plant Syst. Evol..

[13] Friesen N., Smirnov S.V., Shmakov A.I., Herden T., Oyuntsetseg B., Hurka H. (2020). *Allium* species of section *Rhizomatosa*, early members of the Cenrtal Aisan steppe vegetation. Flora.

[14] Bothmer R.V. (1974). Studies in the Aegean Flora. XXI. Biosystematic studies in the *Allium ampeloprasum* complex. Opera Bot..

[15] De Wilde-Duyfjes B.E.E. (1976). A revision of the genus *Allium*, L. (Liliaceae) in Africa. Meded. Landbouwhogesch. Wagening..

[16] Pastor J. (1981). Contribuciòn al estudio de las semillas de las especies de *Allium* de la Peninsula Ibèricae islas Baleares. Lagascalia.

[17] Kruse J. (1984). Rasterelektronenmikroskopische Untersuchungen an Samen der Gattung *Allium* L. Die Kult..

[18] Kruse J. (1986). Rasterelektronenmikroskopische Untersuchungen an Samen der Gattung *Allium* L. II. Die Kult..

[19] Kruse J. (1988). Rasterelektronenmikroskopische Untersuchungen an Samen der Gattung *Allium* L. III. Die Kult..

[20] Kruse J. (1994). Rasterelektronenmikroskopische Untersuchungen an Samen der Gattung *Allium* L. IV. Feddes Repert..

[21] Cesmedziev I., Terzijski D. (1997). A scanning electron microscopic study of the spermoderm in *Allium* subg. *Codonoprasum* (Alliaceae). Bocconea.

[22] Ilarslan H., Koyuncu M. (1997). Türkiye’de yetisen bazi endemic *Allium* (soğan) türlerinin tohum morfolojileri. Ot. Sist. Bot. Derg..

[23] Fritsch R.M., Kruse J., Adler K., Rutten T. (2006). Testa sculptures in *Allium* L. subg. *Melanocrommyum* (Webb and Berth.) Rouy (Alliaceae). Feddes Reper..

[24] Neshati F., Fritsch R.M. (2009). Seed characters and testa sculptures of some Iranian *Allium* L. species (Alliaceae). Feddes Reper..

[25] Choi H.J., Cota-Sanchez J.H. (2010). A taxonomic revision of *Allium* (Alliaceae) in the Canadian prairie provinces. Botany.

[26] Choi H.J., Oh B.U. (2011). A partial revision of *Allium* (Amaryllidaceae) in Korea and northeastern China. Bot. J. Linn. Soc..

[27] Bednorz L., Krzymińska A., Czarna A. (2011). Seed morphology and testa sculptures of some *Allium* L. species (Alliaceae). Acta Agrobot..

[28] Celep F., Koyuncu M., Fritsch R.M., Kahraman A., Doğan M. (2012). Taxonomic importance of seed morphology in *Allium* (Amaryllidaceae). Syst. Bot..

[29] Duman H., Ekşi G., Özbek F. (2017). Two new species *Allium* L. sect *Allium* (Amaryllidaceae) from Turkey. Plant Syst. Evol..

[30] Choi H.J., Giussani L.M., Jang C.G., Oh B.U., Cota-Sanchez J.H. (2012). Systematics of disjunct northeastern Asian and northern North American *Allium* (Amaryllidaceae). Botany.

[31] Lin C.Y., Tan D.Y. (2017). Seed testa micromorphology of thirty-eight species of *Allium* (Amaryllidaceae) from central Asia, and its taxonomic implications. Nord. J. Bot..

[32] Veiskarami G., Khodayari H., Heubl G., Zarre S. (2018). Seed surface ultrastructure as an efficient tool for species delimitation in the *Allium ampeloprasum* L. alliance (Amaryllidaceae, Allioideae). Microsc. Res. Tech..

[33] Deniz İ.G., Genç İ., Sari D. (2015). Morphological and molecular data reveal a new species of *Allium* (Amaryllidaceae) from SW Anatolia, Turkey. Phytotaxa.

[34] Rola K. (2014). Cell pattern and ultrasculpture of bulb tunic of selected *Allium* species (Amaryllidaceae) and their diagnostic value. Acta Biol. Cracov. Bot..

[35] Khorasani M., Saedi Mehrvarz S.h., Zarre S. (2018). Bulb tunic anatomy and its taxonomic implication in *Allium* L. (Amaryllidaceae: Allioideae). Plant Biosyst. Inter. J. Deal. All Asp. Plant Biol..

[36] Khorasani M., Saedi Mehrvarz S., Zarre S. (2018). Scape anatomy and its systematic implication in *Allium stipitatum* species complex (Amaryllidaceae). Nord. J. Bot..

[37] Friesen N. (1995). The genus *Allium* L. in the flora of Mongolia. Feddes Reper..

[38] Lazkov G.A., Sultanova B.A. (2011). Checklist of vascular plants of Kyrgyzstan. Norrlinia.

[39] Sennikov A.N., Lazkov G.A. (2013). *Allium formosum* Sennikov and Lazkov (Amaryllidaceae), a new species from Kyrgyzstan. Phytokeys.

[40] Oyuntsetseg B., Darikhand N., Friesen N. (2013). *Allium carolinianum* DC. A new species to the Outer Mongolia. Turczaninowia.

[41] Urgamal M., Oyuntsetseg B., Nyambayar D., Dulamsuren C. (2014). Conspectus of the Vascular Plants of Mongolia.

[42] Sennikov A.N., Tojibaev K.S., Khassanov F.O., Beshko N.Y. (2016). The flora of Uzbekistan project. Phytotaxa.

[43] Filimonova Z.N. (1971). Morfologija semjan sredneaziatskikh vidovr. *Allium* L. Introd. I Akklim. Rast Na Ukr..

[44] Brullo S., Pavone P., Salmeri C. (2013). *Allium aetnense* (Amaryllidaceae), a new species from Sicily. Plant Biosyst..

[45] Deniz İ.G., Genç İ., Ince A.G., Aykurt C., Elmasulu S., Sümbül H., Sönmez S., Citak S. (2013). Taxonomic data supporting differences between *Allium elmaliense* and *Allium cyrilli*. Biologia.

[46] Genç İ., Ozhatay F.N. (2014). *Allium efeae* (Amaryllidaceae), a new species from northwest Anatolia, Turkey. Turk. J. Bot..

[47] Özhatay N., Koçyigit M., Brullo S., Salmeri C. (2018). *Allium istanbulense*, a new autumnal species of A sect *Codonoprasum* (Amaryllidaceae) from Turkey and its taxonomic position among allied species. Phytotaxa.

[48] Yildirim H. (2019). *Allium sultanae-ismailii* (Amaryllidaceae), a new species from eastern Turkey. Phytotaxa.

[49] Khassanov F.O., Shigyo M., Khar A., Abdelrahman M. (2018). Taxonomical and Ethnobotanical Aspects of *Allium* Species from Middle Asia with Particular Reference to Subgenus *Allium*. The Allium Genomes.

[50] Barthlott W. (1981). Epidermal and seed surface characters of plants: Systematic applicability and some evolutionary aspects. Nord. J. Bot..

[51] Koch K., Bhushan B., Barthlott W. (2009). Multifunctional surface structures of plants: An inspiration for biomimetics. Prog. Mater. Sci..

