# Supplementary material for: Seed Morphology of Allium L. (Amaryllidaceae) from Central Asian Countries and Its Taxonomic Implications
Source: Plants (Basel). 2020 Sep 20;9(9):1239. doi: 10.3390/plants9091239 (PMC7570352; doi:10.3390/plants9091239)
Supplement: Supplementary file 1 [file plants-09-01239-s001.pdf]

plants

Seed morphology of *Allium* L. (Amaryllidaceae) from central Asian countries and its taxonomic implications

Shukherdorj Baasanmunkh[1,†] , Jae Kyoung Lee[1,†], Ju Eun Jang[1], Min Su Park[2], Nikolai Friesen[3], Sungwook Chung[4] and Hyeok Jae Choi[1,*]

[1] Department of Biology and Chemistry, Changwon National University, Changwon, 51140, Korea; Baasanmunkh.sh@gmail.com (S.B.); nosejk@naver.com (J.K.L.); jueunjang222@gmail.com (J.E.J.); hjchoi1975@changwon.ac.kr (H.J.C.)

[2] Department of Biology Education, Kongju National University, Gongju, 32588, Korea; lacmyo14@gmail.com (M.S.P.)

[3] Botanical Garden of the University of Osnabrueck, Osnabrueck, 49076, Germany; friesen@biologie.uni-osnabrueck.de (N.F.)

[4] Department of Computer Engineering, Changwon National University, Changwon, 51140, Korea; swchung@changwon.ac.kr (S.C.)

* Correspondence: hjchoi1975@changwon.ac.kr; Tel.: +82-55-213-3457 (H.J.C.)

† These authors contributed equally to this work.

Table S1. Macro- and micro-morphological characteristics of seed in *Allium* species investigated. The asterisk (*) indicates the first known taxon from this study.

Taxon (voucher code)	Seed length (mm) Mean±SD (min–max)	Seed width (mm) Mean±SD (min–max)	L/W ratio Mean±SD	Seed shape	Seed testa		Figure
					Anticlinal walls	Periclinal walls	
**subg. *Allium***							
**sect. *Allium***							
A. filidens* (100)	**3.27±0.24 (2.76–3.80)**	**2.33±0.18 (1.94–2.71)**	**1.41±0.12**	Oval-flattened	U-type	Some central verrucae and marginal verrucae	1A, 5A
sect. *Caerulea*							
A. caesium (101a)	2.81±0.11 (2.56–3.01)	1.36±0.11 (1.20–1.58)	2.08±0.15	Elliptical-angular	U-type	Densely prominent granules	1B, 5B
A. caesium (101b)	1.88±0.11 (1.69–2.07)	1.34±0.09 (1.15–1.51)	1.41±0.11	Oval-angular	U-, Ω-type	Several large verrucae with small verrucae	1C, 5C
A. caeruleum (102)	2.40±0.10 (2.19–2.61)	1.65±0.09 (1.48–1.85)	1.46±0.09	Oval-angular	U-, Ω-type	Central verrucae and marginal verrucae	1D, 5D
A. svetlanae (103)*	2.59±0.29 (2.28–2.93)	2.12±0.14 (1.95–2.31)	1.22±0.07	Oval-spherical	U-type	Several verrucae and marginal verrucae	1E, 5E
A. tatyanae (104)*	2.04±0.21 (1.67–2.54)	1.13±0.09 (0.96–1.32)	1.81±0.14	Oval-angular	Irregularly-curved	Densely prominent granules	1F, 5F
sect. *Mediasia*							
A. turkestanicum (105)*	3.49±0.30 (2.96–4.06)	2.94±0.40 (2.33–3.65)	1.20±0.12	Oval-spherical	S-type	Large verrucae	1G, 5G
sect. *Minuta*							
A. anisotepalum (106)*	2.10±0.08 (1.89–2.26)	1.06±0.08 (0.83–1.15)	2.00±0.15	Elliptical-flattened	Irregularly-curved	Many small verrucae with granules	1H, 5H
sect. *Pallasia*							
A. pallasii (107)	2.89±0.12 (2.65–3.09)	1.75±0.10 (1.53–2.02)	1.66±0.11	Oval-angular	U-type	Densely prominent granules	1I, 5I
subg. *Butomissa*							
sect. *Austromotana*							
A. oreoprasum (108)	4.08±0.23 (3.26–4.41)	2.11±0.12 (1.93–2.44)	1.94±0.14	Oval-angular	Irregularly-curved	Many small verrucae with marginal granules	1J, 5J
sect. *Butomissa*							
A. ramosum (109)	3.42±0.23 (2.89–3.78)	2.71±0.24 (2.16–3.03)	1.27±0.10	Oval-hemispherical	Irregularly-curved to nearly-straight	Densely granules	1K, 5K
**subg. *Cepa***							

sect. *Annuloprason*							
A. fedschenkoanum (110)*	4.47±0.43 (3.63–5.49)	1.99±0.29 (1.50–2.82)	2.27±0.29	Elliptical-angular	Straight	Colliculose cellular center and verrucae	1L, 5L
sect. *Cepa*							
A. altaicum (111)*	3.51±0.12 (3.22–3.75)	2.23±0.12 (1.92–2.46)	1.57±0.07	Oval-angular	Straight	Densely granules	1M, 5M
A. galanthum (112)	4.03±0.10 (3.72–4.17)	2.33±0.09 (2.08–2.49)	1.74±0.06	Oval-angular	Straight	Densely granules	1N, 5N
A. oschaninii (113)	3.86±0.17 (3.56–4.26)	2.49±0.13 (2.28–2.81)	1.56±0.08	Oval-angular	Straight	Several verrucae and marginal small verrucae	1O, 5O
sect. *Schoenoprasum*							
A. maximowiczii (114)	2.23±0.09 (2.02–2.37)	1.12±0.07 (0.98–1.28)	2.01±0.11	Elliptical-angular	Straight	Several small verrucae with densely granules	1P, 6A
**subg. *Melanocrommyum***							
sect. *Acmopetala*							
A. saposhnikovii (115)	2.72±0.12 (2.51–2.95)	1.80±0.10 (1.56–1.94)	1.52±0.07	Oval-angular	U-, Ω-type	A central verrucate verruca or rarely few verrucae	2A, 6B
sect. *Kaloprason*							
A. alexeianum (116)	2.56±0.23 (2.24–3.12)	2.12±0.21 (1.71–2.56)	1.22±0.08	Oval-hemispherical	U-, Ω-type	A large central verruca	2B, 6C
A. caspium(117)	2.66±0.16 (2.34–3.15)	2.36±0.22 (1.96–2.89)	1.14±0.11	Oval-hemispherical	U-, Ω-type	Large verrucate verrucae	2C, 6D
A. protensum (118)	3.05±0.17 (2.65–3.52)	2.53±0.23 (2.02–3.12)	1.21±0.13	Oval-spherical	U-, Ω-type	A big central verrucate verruca and marginal small verrucae	2D, 6E
sect. *Miniprason*							
A. karataviense (119)	4.36 ± 0.19 (4.01–4.89)	3.44 ± 0.23 (3.05–3.88)	1.27±0.07	Oval-hemispherical	S-, U-type	Prominent verrucae	2E, 6F
sect. *Procerallium*							
A. altissimum (120)	2.98±0.21 (2.66–3.74)	2.65±0.18 (2.35–3.22)	1.13±0.06	Oval-spherical	S-type	Several big verrucae	2F, 6G
A. stipitatum (121)	3.84±0.39 (3.01–4.62)	2.96±0.26 (2.47–3.50)	1.30±0.12	Oval-hemispherical	U-, Ω-type	Several big verrucae	2G, 6H
sect. *Stellata*							
A. taeniopetalum (122)*	3.38±0.21 (2.83–4.06)	2.62±0.18 (2.32–2.95)	1.30±0.11	Oval-hemispherical	U-, Ω-type	Several prominent verrucae	2H, 6I
sect. *Verticillata*							
A. viridiflorum (123)	1.89±0.17 (1.58–2.23)	1.45±0.10 (1.22–1.61)	1.31±0.09	Oval-hemispherical	Irregularly-curved	Densely prominent granules	2I, 6J
**subg. *Polyprason***							

sect. *Falcatifolia*							
A. carolinianum (124)	3.78±0.11 (3.59–4.01)	1.87±0.16 (1.53–2.16)	2.03±0.18	Elliptical-angular	Straight	Densely indistinct granules	2J, 6K
A. hymenorrhizum (125)	3.77±0.24 (3.45–4.50)	1.95±0.11 (1.69–2.22)	1.93±0.15	Oval-flattened	Straight	A central verruca and several marginal small verrucae	2K, 6L
A. platyspathum subsp. *amblyophyllum* (126a)	3.30±0.14 (2.97–3.58)	1.98±0.21 (1.49–2.35)	1.69±0.16	Oval-angular	Straight	Densely indistinct granules	2L, 6M
A. platyspathum subsp. *amblyophyllum* (126b)	3.84±0.12 (3.63–4.04)	2.09±0.11 (1.70–2.25)	1.84±0.10	Oval-angular	Straight	Densely indistinct granules	6N
A. platyspathum subsp. *platyspathum* (127)	3.58±0.34 (3.14–4.24)	1.90±0.14 (1.62–2.14)	1.89±0.20	Oval-angular	Straight	Densely indistinct granules	2M, 6O
A. korolkowii (128)	2.82±0.16 (2.46–3.07)	1.44±0.14 (1.07–1.62)	1.98±0.16	Oval-angular	Straight	Small verrucae	2N, 7A
sect. *Oreiprason*							
A. obliquum (129)	3.61±0.17 (3.27–3.97)	1.73±0.11 (1.53–1.91)	2.09±0.12	Elliptical-angular	Straight	Small verrucae with densely granules	2O, 7C
A. petraeum (130)*	3.27±0.17 (2.92–3.55)	1.80±0.13 (1.47–2.03)	1.83±0.17	Oval-angular	Straight	Several granules	2P, 7D
A. tianschanicum (131)	3.91±0.12 (3.60–4.08)	2.27±0.16 (1.89–2.90)	1.73±0.10	Oval-angular	Straight	Indistinct verrucae or granules	2Q, 7E
A. kirilovii (132)*	3.08±0.52 (2.59–3.94)	1.50±0.14 (1.24–1.67)	2.08±0.44	Elliptical-angular	Straight	Densely prominent granules	3A, 7B
**subg. *Reticulatobulbosa***							
sect. *Campanulata*							
A. barsczewskii (133)	3.87±0.15 (3.49–4.07)	1.74±0.09 (1.54–1.88)	2.23±0.10	Elliptical-flattened	Straight	A central verrucate verruca and marginal verrucae	3B, 7F
A. dolichostylum (134)*	3.69±0.18 (3.29–3.98)	1.96±0.08 (1.87–2.17)	1.88±0.08	Oval-flattened	Straight	A central verrucate verruca and marginal small verrucae	3C, 7G
A. jodanthum (135)*	3.93±0.25 (3.43–4.31)	1.85±0.14 (1.63–2.17)	2.13±0.18	Elliptical-flattened	Straight	Granulate verrucae and several prominent granules	3D, 7H
sect. *Reticulatobulbosa*							
A. amphibolum (136a)*	3.54±0.11 (3.27–3.84)	1.64±0.08 (1.43–1.77)	2.16±0.12	Elliptical-angular	Straight	Densely granules	3E, 7I
A. amphibolum (136b)*	3.44±0.12 (3.22–3.81)	1.45±0.07 (1.41–1.74)	2.14±0.12	Elliptical-angular	Straight	Usually a granulate central verruca and marginal	7J

						granules	
A. clathratum (137)*	3.29±0.27 (2.79–3.93)	1.43±0.10 (1.24–1.67)	2.31±0.20	Elliptical-angular	Straight	Usually a granulate central verruca and marginal granules	3F, 7K
A. leucocephalum (138)*	2.72±0.17 (2.47–3.07)	1.30±0.08 (1.16–1.54)	2.09±0.13	Elliptical-angular	Straight	Densely granules	3G, 7L
A. malyschevii (139)*	3.46±0.23 (3.00–3.77)	1.58±0.18 (1.24–1.87)	2.22±0.24	Elliptical-angular	Straight	Several granulate verrucae, densely granules	3H, 7M
A. strictum (140)	3.12±0.21 (2.39–3.41)	1.47±0.09 (1.27–1.68)	2.12±0.15	Elliptical-angular	Straight	A central large granulate verruca and densely granules	3I, 7N
sect. *Scabriscapa*							
A. trachyscordum (141)*	3.18±0.18 (2.92–3.93)	2.35±0.14 (2.05–2.75)	1.36±0.08	Oval-angular	Straight	A central large verruca and marginal small verrucae	3J, 7O
**subg. *Rhizirideum***							
sect. *Caespitosoprason*							
A. bidentatum (142)	2.45±0.21 (1.99–2.85)	1.63±0.17 (1.31–1.87)	1.51±0.12	Oval-angular	Straight	Densely prominent granules	3K, 8A
A. polyrhizum (143)	2.90±0.14 (2.54–3.16)	1.74±0.16 (1.48–2.02)	1.67±0.14	Oval-angular	Straight	Densely indistinct granules	3L, 8B
sect. *Rhizirideum*							
A. austrosibiricum (144)*	2.63±0.15 (2.11–2.96)	1.72±0.12 (1.54–2.04)	1.54±0.13	Oval-hemispherical	Straight	Densely prominent granules	3M, 8C
sect. *Tenuissima*							
A. anisopodium (145)	2.39±0.10 (2.20–2.62)	1.55±0.10 (1.33–1.72)	1.55±0.11	Oval-angular	S-type	Densely granules	3N, 8D
A. tenuissimum (146)	1.74±0.16 (1.56–1.94)	1.34±0.14 (1.20–1.58)	1.31±0.13	Oval-angular	S-type	Densely granules	3O, 8E
A. vodopjanovae (147)	2.36±0.22 (1.85–2.76)	1.53±0.19 (1.19–1.96)	1.56±0.15	Oval-angular	S-type	Densely granules	3P, 8F

Table S2. Voucher specimen information of *Allium* species investigated. All voucher specimens were deposited at the herbarium of Changwon National University.

Subgenus/Section	Taxon	Voucher information
Allium/Allium	*A. filidens* Regel	Uzbekistan: Chatkal Mountain Shavassay, 16 Jul 2016, *H.J. Choi et al. 100*
Allium/Caerulea	*A. caesium* Schrenk	Uzbekistan: Tashkent Botanical Garden, 26 May 2015, *H.J. Choi et al. 101a*
Allium/Caerulea	*A. caesium*	Kyrgyzstan: Gornolyzhnyy Kurort "Politekh", 02 Aug 2017, *H.J. Choi et al. 101b*
Allium/Caerulea	*A. caeruleum* Pall.	Uzbekistan: ZX10, Tashkent Botanical Garden, 14 Jul 2017, *H.J. Choi et al. 102*
Allium/Caerulea	*A. svetlanae* Vved. ex Filim	Uzbekistan: Samarkand region, Naratau, 07 Jul 2016, *H.J. Choi et al. 103*
Allium/Caerulea	*A. tatyanae* F.O. Khass. & F. Karimov	Uzbekistan: Namangan city, Yangikurgan, Hill of Ungoe, 24 May 2015, *H.J. Choi et al. 104*
Allium/Mediasia	*A. turkestanicum* Regel	Uzbekistan: Nuratau region, Tashkent Botanical Garden 3/G/5, 28 Aug 2014, *H.J. Choi et al. 105*
Allium/Minuta	*A. anisotepalum* Vved.	Uzbekistan: Namangan city, Yangikurgan, Hill of Ungoe, 24 May 2015, *H.J. Choi et al. 106*
Allium/Pallasia	*A. pallasii* Murray	Kyrgyzstan: Kara-balta river, 14 Jul 2017, *H.J. Choi et al. 107*
Butomissa/Austromotana	*A. oreoprasum* Schrenk	Kyrgyzstan: 7 km north-northwestward from Too-Ashuu, 04 Aug 2017, *H.J. Choi et al. 108*
Butomissa/Butomissa	*A. ramosum* L.	Mongolia: Tuv province, Hustai National Park , 11 Aug 2016, *H.J. Choi et al. 109*
Cepa/Annuloprason	*A. fedschenkoanum* Regel	Kyrgyzstan: 2 km northwest side from Baytur Resort, 02 Aug 2017, *H.J. Choi et al. 110*
Cepa/Cepa	*A. altaicum* Pall.	Mongolia: Khovd province, Munkhkhairkhan, Senkheriin khavtsal, 25 Jul 2016, *H.J. Choi et al. 111*
Cepa/Cepa	*A. galanthum* Kar. & Kir.	Kyrgyzstan: 2 km west from Krasnyy Village, 03 Aug 2017, *H.J. Choi et al. 112*
Cepa/Cepa	*A. oschaninii* O. Fedtsch.	Uzbekistan: Tashkent Botanical Garden, 14 Jul 2017, *H.J. Choi et al. 113*
Cepa/Schoenoprasum	*A. maximowiczii* Regel	Mongolia: Dornod province, Khalkh gol Sum, Sumiin Khooloi, 18 Jul 2017, *H.J. Choi et al. 114*
Melanocrommyum/Acmopetala	*A. saposhnikovii* Nikitina	Kyrgyzstan: 7km south side of Jergalan, 30 Jun 2018, *H.J. Choi et al. 115*
Melanocrommyum/Kaloprason	*A. alexeianum* Regel	Uzbekistan: Qashqadaryo province, Qamashi Tumani, Majdanak, 05 Sept 2015, *H.J. Choi et al. 116*
Melanocrommyum/Kaloprason	*A. caspium* (Pall.) M. Bieb.	Uzbekistan: Tashkent Botanical Garden (S14), 17 Jun 2017, *H.J. Choi et al. 117*
Melanocrommyum/Kaloprason	*A. protensum* Wendelbo	Uzbekistan: Nurate, 11 May 2015, *H.J. Choi et al. 118*
Melanocrommyum/Miniprason	*A. karataviense* Regel	Uzbekistan: Yangiabed Ferganya Valley, 11 May 2015, *H.J. Choi et al. 119*
Melanocrommyum/Procerallium	*A. altissimum* Regel	Uzbekistan: Nurati vit, 11 May 2015, *H.J. Choi et al. 120*
Melanocrommyum/Procerallium	*A. stipitatum* Regel	Uzbekistan: Qashqadaryo province, Rode (M39), 05 Sep 2015, *H.J. Choi et al. 121*
Melanocrommyum/Stellata	*A. taeniopetalum* Popov & Vved.	Uzbekistan: Jizzakh Pistali tau, 11 May 2015, *H.J. Choi et al. 122*
Melanocrommyum/Verticilata	*A. viridiflorum* Pobed.	Uzbekistan: Yangikurgan region Pozamon Chatkal range, 14 Jul 2017, *H.J. Choi et al. 123*
Polyprason/Falcatifolia	*A. carolinianum* DC.	Kyrgyzstan: Kara-balta river, 06 Jul 2016, *H.J. Choi et al. 124*
Polyprason/Falcatifolia	*A. hymenorrhizum* Ledeb.	Kyrgyzstan: 1 km south side from Baytur Resort, 01 Aug 2017, *H.J. Choi et al. 125*
Polyprason/Falcatifolia	*A. platyspathum* subsp. *amblyophyllum* (Kar. & Kir.) N. Friesen	Kyrgyzstan: 4 km east side from Baytur Resort, 01 Aug 2017, *H.J. Choi et al. 126a*
Polyprason/Falcatifolia	*A. platyspathum* subsp.	Mongolia: Bayan-Ulgii province, Sagsai sum, Songiniin Gol, 18 Jun 2018, *H.J. Choi et al. 126b*

	amblyophyllum	
Polyprason/Falcatifolia	*A. platyspathum* subsp. *platyspathum*	Kyrgyzstan: 13 km south side from Jergalan, 30 Jun 2018, *H.J. Choi et al. 127*
Polyprason/Falcatifolia	*A. korolkowii* Regel	Uzbekistan: Cholpon Ata, 10 Jul 2016, *H.J. Choi et al. 128*
Polyprason/Oreiprason	*A. kirilovii* N. Friesen & Seregin	Kyrgyzstan: 2 km northwest side from Baytur Resort, 03 Aug 2017, *H.J. Choi et al. 129*
Polyprason/Oreiprason	*A. obliquum* L.	Kyrgyzstan: the Narin mountain, north makrosclone, Narin NES, 10 Aug 2014, *G.A. Lazkov 130*
Polyprason/Oreiprason	*A. petraeum* Kar. & Kir.	Kyrgyzstan: Gornolyzhnyy Kurort "Politekh" 02 Aug 2017, *H.J. Choi et al. 131*
Polyprason/Oreiprason	*A. tianschanicum* Rupr.	Kyrgyzstan: 7km north-northwestward from Too-Ashuu, 04 Aug 2017, *H.J. Choi et al. 132*
Reticulatobulbosa/Campanulata	*A. barsczewskii* Lipsky	Uzbekistan: Yangikurgan region Pozamon Chatkal range, 14 Jul 2017, *H.J. Choi et al. 133*
Reticulatobulbosa/Campanulata	*A. dolichostylum* Vved.	Kyrgyzstan: Sary-chclek, Chatkal range, 11 Aug 2016, *G.A. Lazkov 134*
Reticulatobulbosa/Campanulata	*A. jodanthum* Vved.	Uzbekistan: Yangiabed, 02 Jul 2016, *H.J. Choi et al. 135*
Reticulatobulbosa/Reticulatobulbosa	*A. amphibolum* Ledeb.	Mongolia: Khovd province, Munkhkhairkhan, Senkheriin khavtsal, 25 Jul 2016, *Sh. Baasanmunkh et al. 136a*
Reticulatobulbosa/Reticulatobulbosa	*A. amphibolum*	Mongolia: Bayan-Ulgii province, Sagsai sum, Songiniin Gol, 18 Jun 2018, *Sh. Baasanmunkh et al. 136b*
Reticulatobulbosa/Reticulatobulbosa	*A. clathratum* Ledeb.	Mongolia: Khovd province, Munkhkhairkhan, Senkheriin khavtsal, 27 Jul 2016, *Sh. Baasanmunkh et al. 137*
Reticulatobulbosa/Reticulatobulbosa	*A. leucocephalum* Turcz. ex Ledeb.	Mongolia: Tuv province, Hustai National Park, 11 Aug 2016, *Sh. Baasanmunkh et al. 138*
Reticulatobulbosa/Reticulatobulbosa	*A. malyschevii* N. Friesen	Mongolia: Khuvsgul province, Khatgal sum, Khuvsgul Lake, 15 Jul 2015, *Sh. Baasanmunkh et al. 139*
Reticulatobulbosa/Reticulatobulbosa	*A. strictum* Schrad.	Mongolia: Khuvsgul province, Khatgal sum, Khuvsgul Lake, 17 Jul 2015, *Sh. Baasanmunkh et al. 140*
Reticulatobulbosa/Scabriscapa	*A. trachyscordum* Vved.	Kyrgyzstan: 35 km southeast side from Novovoznesenovka, 30 Jun 2018, *H.J. Choi et al. 141*
Rhizirideum/Caespitosoprason	*A. bidentatum* Fisch. ex Prokh.	Mongolia: Tuv province, Hustai National Park, 11 Aug 2016, *H.J. Choi et al. 142*
Rhizirideum/Caespitosoprason	*A. polyrhizum* Turcz. ex Regel	Mongolia: Govisumber province, Shivee-Ovoo, 21 Aug 2016, *Sh. Baasanmunkh et al. 143*
Rhizirideum/Rhizirideum	*A. austrosibiricum* N. Friesen	Mongolia: Khovd province, Munkhkhairkhan, Khuren khesuu, 30 Jul 2016, *H.J. Choi et al. 144*
Rhizirideum/Tenuissima	*A. anisopodium* Ledeb.	Mongolia: Khovd province, Mankhan sum, 3 tsenkheriin agui, 31 Jul 2016, *H.J. Choi et al. 145*
Rhizirideum/Tenuissima	*A. tenuissimum* L.	Mongolia: Khentii province, 25 km from Berkh sum, 11 Jul 2017, *H.J. Choi et al. 146*
Rhizirideum/Tenuissima	*A. vodopjanovae* N. Friesen	Mongolia: Khovd province, Munkhkhairkhan, Tsagaan sair, 30 Jul 2016, *H.J. Choi et al. 147*
